# TNF-α Mediates Eosinophil Cationic Protein-induced Apoptosis in BEAS-2B Cells

**DOI:** 10.1186/1471-2121-11-6

**Published:** 2010-01-20

**Authors:** Kun-Che Chang, Chih-Wei Lo, Tan-chi Fan, Margaret Dah-Tsyr Chang, Chih-Wen Shu, Chuan-Hsin Chang, Cheng-Ta Chung, Shun-lung Fang, Chih-Chung Chao, Jaw-Ji Tsai, Yiu-Kay Lai

**Affiliations:** 1 Institute of Biotechnology & Department of Life Science, National Tsing Hua University, Hsinchu, 30013 Taiwan; 2 Institute of Molecular and Cellular Biology & Department of Life Science, National Tsing Hua University, Hsinchu, 30013 Taiwan; 3 Department of Allergy and Clinical Immunology, Taichung Veterans' General Hospital, Taichung, 40705 Taiwan; 4 Department of Bioresources, Da-Yeh University, Changhua, 51591 Taiwan

## Abstract

**Background:**

Eosinophilic granulocytes are important for the human immune system. Many cationic proteins with cytotoxic activities, such as eosinophil cationic protein (ECP) and eosinophil-derived neurotoxin (EDN), are released from activated eosinophils. ECP, with low RNase activity, is widely used as a biomarker for asthma. ECP inhibits cell viability and induces apoptosis to cells. However, the specific pathway underlying the mechanisms of ECP-induced cytotoxicity remains unclear. This study investigated ECP-induced apoptosis in bronchial epithelial BEAS-2B cells and elucidated the specific pathway during apoptosis.

**Results:**

To address the mechanisms involved in ECP-induced apoptosis in human BEAS-2B cells, investigation was carried out using chromatin condensation, cleavage of poly (ADP-ribose) polymerase (PARP), sub-G1 distribution in cell cycle, annexin V labeling, and general or specific caspase inhibitors. Caspase-8-dependent apoptosis was demonstrated by cleavage of caspase-8 after recombinant ECP treatment, accompanied with elevated level of tumor necrosis factor alpha (TNF-α). Moreover, ECP-induced apoptosis was effectively inhibited in the presence of neutralizing anti-TNF-α antibody.

**Conclusion:**

In conclusion, our results have demonstrated that ECP increased TNF-α production in BEAS-2B cells and triggered apoptosis by caspase-8 activation through mitochondria-independent pathway.

## Background

Eosinophilic granulocytes, commonly called eosinophils, are leukocytes that develop in the bone marrow and differentiate from hematopoietic progenitor cells [1]. Eosinophils traffic into tissues, such as the gastrointestinal, genitourinary and respiratory tracts [2], and are recruited to airway tissues during the asthmatic inflammatory process [3]. Activated eosinophils release cytokines such as tumor necrosis factor alpha (TNF-α) [1] and granular toxic proteins. Among which eosinophil cationic protein (ECP) and eosinophil-derived neurotoxin (EDN) share 67% amino acid sequence identity [4] and play important roles in the pathogenesis of mammalian cells [5].

ECP is a member of the pancreatic-type extracellular ribonuclease (RNase) family, in which ECP and EDN are respectively named as RNase3 and RNase2 [6]. It has been extensively investigated as an efficacious biomarker of airway inflammation such as asthma [7] and has been suggested as a causal factor in allergic respiratory disease [8]. ECP is a potent cytotoxic protein capable of killing cells of guinea pig tracheal epithelium [9], mammalian leukemia [10], epidermis carcinoma [9], and breast carcinoma [11] as well as non-mammalian cells such as parasites, bacteria, and viruses [12]. The molecular mechanisms of ECP cytotoxicity are not involved in its RNase activity [13]. Interestingly, we have previously shown that the signal peptide of ECP is toxic to cells lacking of the signal peptide peptidase, an intra-membrane protease located in the endoplasmic reticulum (ER) [14] and it also triggers up-regulation of transforming growth factor alpha (TGF-α) expression in human cells [15]. Mature ECP devoid of the 27-residue signal peptide contains 133 residues with high positive charges [16]. Cellular uptake and cytotoxicity of RNases have been correlated with the pI value and positive charge [17,18]. We have recently reported that mature ECP is cytotoxic to human bronchial epithelial (BEAS-2B) cells by specific binding to cell surface heparan sulfate proteoglycans (HSPGs) followed by endocytosis [19,20].

Many RNases, such as EDN, Onconase (ONC), and ECP have been reported to induce apoptosis in cells [21-23]. In one such study, a synthetic peptide of EDN was found to induce apoptosis in Kaposi's sarcoma cells [22]. Moreover, ONC, one member of bullfrog RNase A superfamily, displays apoptosis to tumor cells [23]. A latest study indicated that ECP caused cytotoxicity in HL-60 and HeLa cells *via *caspase-3 like activity [21]. Accordingly, cytotoxic RNases play an important role in cell death. However, the mechanism of ECP-induced apoptosis is still not fully verified. Recent studies have shown that eosinophils can induce epithelial cell death *via *apoptosis and necrosis [24]. In addition, apoptosis of airway epithelium cells (AECs) has been reported as a mechanism for removing damaged cells to maintain AEC function such as immune and inflammatory modulators [25,26]. It has also been suggested that AECs in response to different external invasions (e.g., pathogens) can protect themselves [25]. However, the specific apoptosis pathway in ECP-induced human AEC death remains unclear.

Apoptosis, also called programmed cell death, is generally distinguished into two types--caspase-dependent and caspase-independent [27,28]--with the former being the major type. Caspases belong to the cysteinyl aspartate protease family and are classified as effectors (caspases-3, -7, and -6) and initiators (caspases-2, -8, -9, and -10) of programmed cell death. In addition, caspase-12 is reported to be an inflammatory caspase [29]. Currently caspase-dependent apoptosis is divided into three pathways: two intrinsic mitochondria- and ER-associated pathways [30,31] and one extrinsic death receptor-initiated pathway [32]. Mitochondrial membrane potential (MMP) represents a crucial check-point involving caspase-9, which leads to apoptosis [33]. A current study showed that ER stress response involved in caspase-12 could induce apoptosis [34], and consequently the ER stress-induced chaperones such as 78-kDa glucose-regulated protein (GRP78) were activated to rescue the cells. GRP78 inhibits apoptotic signaling through ER or non-ER stress [35]. Caspase-8-dependent apoptosis may be triggered by cell surface receptors belonging to the tumor gene superfamily, including CD95 (or Fas) [36,37], TNF receptor-1 (TNFR1) [38], and TNF-related apoptosis-inducing ligand (TRAIL) [39,40]. Another mechanism for initiating the proteolytic cascade is induced by engagement of TNFR, Fas/APO-1/CD95, triggering caspase-3 activation by activated-caspase-8 without involvement of mitochondria [41,42]. Regarding the ligand of TNFR, TNF-α, it has been reported to be released from epithelial cells [43] and activated eosinophils [44]. Moreover, it is known that poly (ADP-ribose) polymerase (PARP) is cleaved by caspase-3, a downstream caspase of caspase-8, -9, and -12, and causes cell apoptosis [45].

In general, asthma patients have a higher concentration of ECP in serum, bronchoalveolar lavage, and sputum, along with tissue damage than healthy people [46-48]. Severe damage and shedding are commonly observed in asthmatic airway epithelium [49]. Therefore, understanding the mechanism of ECP-induced apoptosis might provide practical methods to treat asthma. Here we intended to determine if BEAS-2B cell death occurred primarily due to apoptosis after ECP treatments, and verified the pathway involved in apoptotic cells.

## Results

### rECP causes cell death and apoptosis

**Figure 1:**
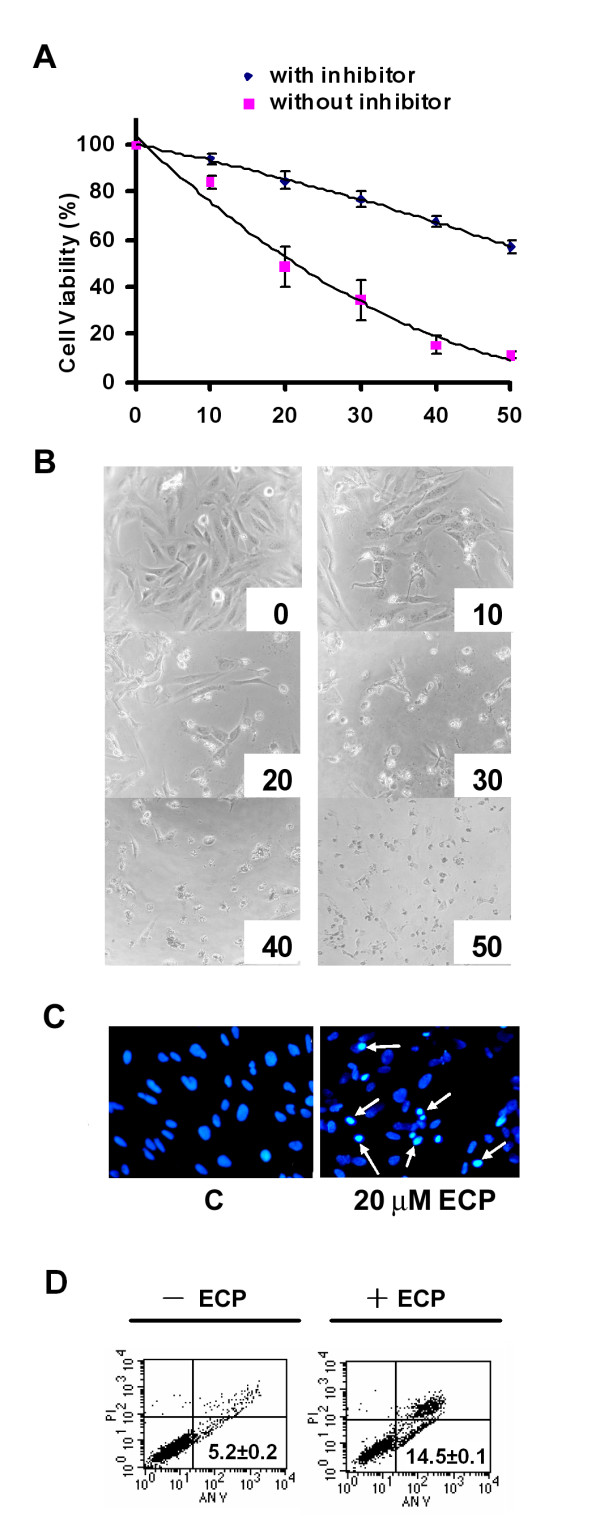
**Effect of rECP on the viability of BEAS-2B cells**. BEAS-2B cells (5 × 10^3^) were incubated in a 96-well plate and treated with various concentrations of rECP as indicated for 48 h. Cell viability was determined by the MTT assay. (**A**) Cells were treated with rECP (up to 50 μM) for 48 h in the presence or absence of the general caspase inhibitor Z-VAD-FMK. (**B**) Morphology of the cells treated with serial concentrations of rECP ranging from 0 to 50 μM (concentrations shown below each panel). (**C**) Nuclei of BEAS-2B cells were stained with Hoechst 33342. Cells were treated or untreated with 20 μM rECP for 48 h. Stained nuclei were visualized by fluorescence microscopy. The chromatin condensation is indicated by bright blue spots shown by white arrows. (**D**) BEAS-2B cells were incubated in the presence or absence of 20 μM rECP for 24 h. The cells were stained with annexin-V-FITC and analyzed by FACS. Intact cells are located in the lower left quadrant. The apoptotic cells stained by annexin-V-FITC are located in the lower right quadrants, respectively. All data represent the arithmetic mean ± SEM.

The effect of rECP on BEAS-2B cell viability was determined by the MTT assay. The rECP was added to the cell culture at concentrations of 0, 10, 20, 30, 40 and 50 μM at 37°C for 48 h. rECP inhibited cell viability with an IC_50 _of 21.03 μM, and cell viability was rescued by general caspase inhibitor, Z-VAD-FMK (Figure 1A). After co-incubation with rECP, shrinkage and unattachment of the cells from culture plate were observed (Figure 1B). BEAS-2B is a human bronchial epithelial cell line which is quite similar to primary cell. To determine whether such cell death was related to apoptosis, the nuclei were stained with Hoechst 33342 to monitor condensation of nuclear chromatin. Bright spots in the rECP-treated cells indicated nuclei undergoing chromatin condensation, strongly suggesting that BEAS-2B cells underwent apoptosis (Figure 1C). Here, apoptosis was also evaluated by staining with annexin V, a reagent commonly used to detect early apoptosis. BEAS-2B cells were treated with 20 μM rECP for 24 h. The treated BEAS-2B cells showed 14.5 ± 0.1% apoptosis (Figure 1D). Besides, the characteristic DNA fragmentation upon treatment with rECP was observed (Additional file 1). In comparison with untreated cells, the data indicated that BEAS-2B cells underwent early apoptosis after treatment with rECP.

### rECP alters cell cycle distribution in BEAS-2B cells

**Figure 2:**
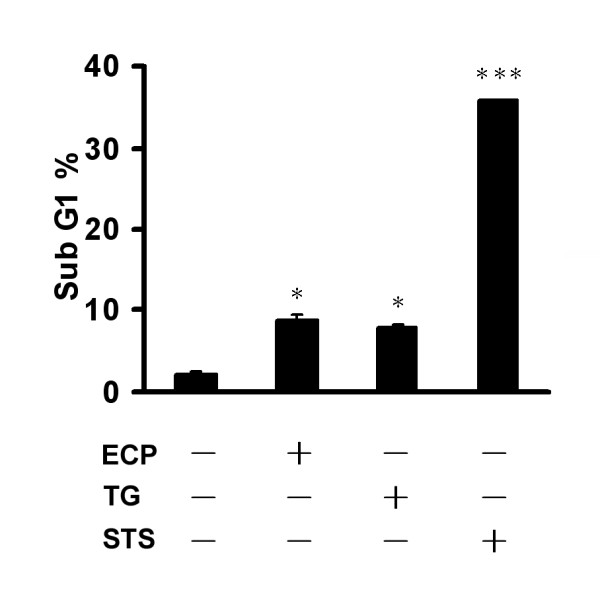
**Effect of rECP, staurosporine, and thapsigargin on cell cycle progression**. BEAS-2B cells were incubated in the presence or absence of 20 μM rECP, 0.1 μM staurosporine (STS) or 1 μM thapsigargin (TG) for 24 h. Cells were stained by PI, and DNA content was analyzed by FACS. The proportion of sub-G1 cells was analyzed in each treatment. All data represent the arithmetic mean ± SEM. *P < 0.05, ***P < 0.001.

DNA damage is a general phenomenon in apoptotic cells and usually determined by sub-G1 cell cycle progression. To investigate whether caspase-9 and -12, markers of mitochondria and ER, respectively, were activated in BEAS-2B cells, specific pathway inducers were used as alternative apoptotic initiators for comparison. BEAS-2B cells were treated separately with 0.1 μM staurosporin (STS), a strong mitochondrial damage inducer, and 1 μM thapsigargin (TG), a strong ER response inducer, for 24 h and stained with PI prior to sub-G1 DNA population analysis employing fluorescent-activated cell sorting (FACS) (Figure 2). The fraction of untreated control cells in sub-G1 was 2%, and that of cells treated with STS, rECP and TG was increased significantly up to 36%, 9% and 7%. Therefore, the increase of sub-G1 fraction in the individual treatments represented the cells undergoing apoptosis. Here TG and STS were able to induce apoptosis in BEAS-2B cells through the ER response and mitochondrial damage pathways, respectively.

### rECP induces apoptosis in a caspase-dependent manner

**Figure 3:**
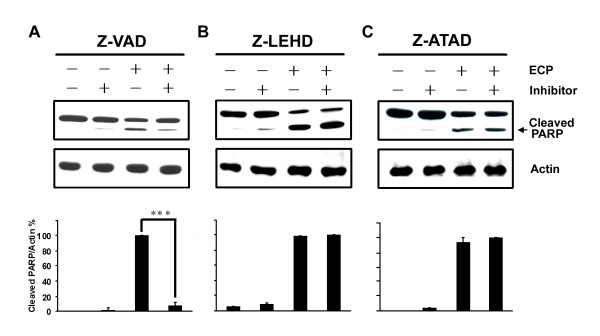
**Effect of caspase inhibitors on rECP-induced apoptosis**. (**A**) To investigate the apoptotic pathway, BEAS-2B cells were pretreated with 20 μM general caspase inhibitor (Z-VAD), respectively, for 30 min before rECP treatment. (**B **and **C**) Cells were treated with 20 μM caspase 9 or 12 inhibitor, Z-LE(OMe)HD(OMe)-FMK (Z-LEHD) or Z-ATAD-FMK (Z-ATAD), respectively, for 30 min before rECP treatment. After 48 h incubation in the presence or absence of 20 μM rECP, total cell lysates were resolved by 10% SDS-PAGE, and relative cleaved PARP was determined by western blotting with anti-PARP and anti-actin followed by quantitative analysis. All data represent the arithmetic mean ± SEM. ***P < 0.001.

In general, activation of the caspase cascade plays an important role in apoptosis. To identify possible involvement of caspases in ECP-induced apoptosis, BEAS-2B cells were treated with rECP in the presence or absence of general caspase inhibitor Z-VAD-FMK and specific caspase-9 and -12 inhibitors, Z-LE(OMe)HD(OMe)-FMK and Z-ATAD-FMK, respectively. The presence of cleaved poly (ADP-ribose) polymerase (PARP) was monitored to evaluate the degree of apoptosis. As compared with the control cells without drug treatment, apoptosis was clearly blocked by caspase inhibitors. The levels of cleaved PARP decreased 92% upon pre-treatment with Z-VAD-FMK (Figure 3A), suggesting that ECP-induced apoptosis proceeded *via *the caspase-dependent pathway. However, cells pre-treated with Z-LE(OMe)HD(OMe)-FMK (Figure 3B) or Z-ATAD-FMK (Figure 3C) exhibited no effect upon treatment with rECP, suggesting that caspase-9 and caspase-12 were not the major pathways involved in cell death triggered by rECP.

### rECP induces ER-independent apoptosis

**Figure 4:**
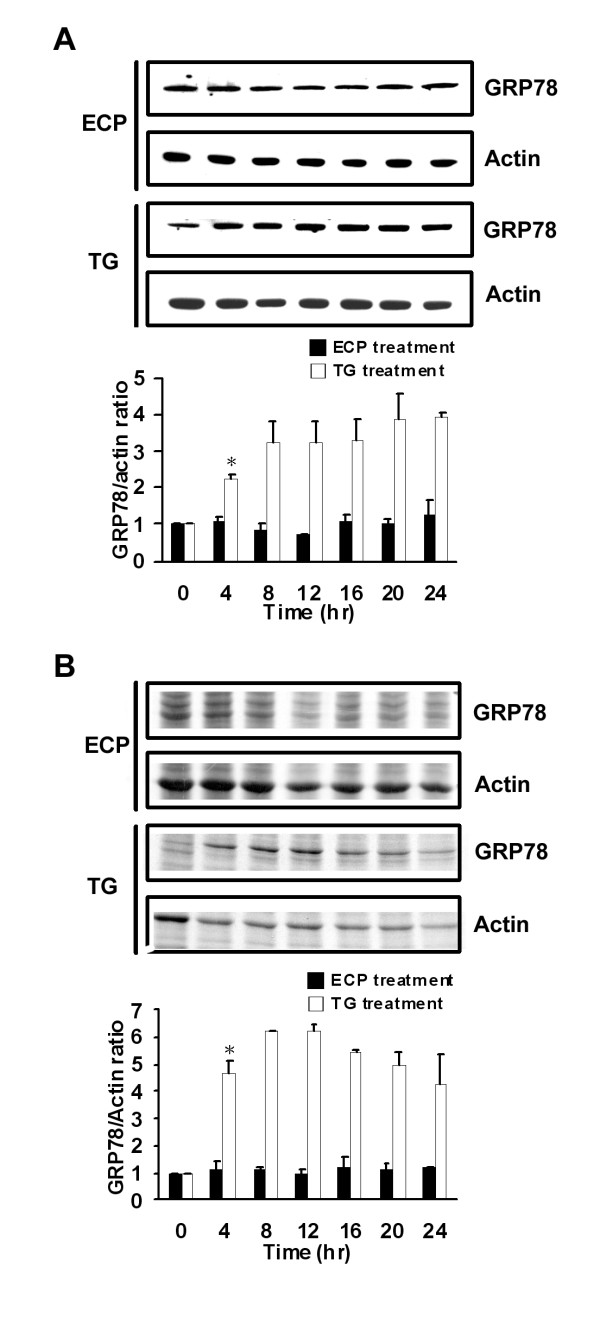
**Effect of rECP-induced apoptosis on ER response**. BEAS-2B cells were treated with rECP or thapsigargin (TG; an ER toxin as a positive control) for the indicated times. The cells used to determine the ER response were incubated with 20 μM rECP or 1 μM TG for 0, 4, 8, 12, 16, 20 and 24 h. (**A**) Total cell lysates were resolved by 10% SDS-PAGE, and the relative accumulated GRP78 was investigated by western blotting with anti-GRP78 and anti-actin. (**B**) For *de novo *proteins synthesis, cells were labeled with [^35^S]methionine for 2 h before harvesting. Equal amounts of labeled cell lysates were resolved by 10% SDS-PAGE followed by quantitative analysis. All data represent the arithmetic mean ± SEM. *P < 0.05.

The ER response is generally triggered by environmental stress and sometimes leads to apoptosis. Because GRP78 plays a crucial role in the ER response [50], the level of GRP78 expression in BEAS-2B cells treated with rECP was assessed by Western blotting and a *de novo *synthesis assay. Accumulated and newly synthesized GRP78 were detected using anti-GRP78 (Figure 4A) and metabolic labeling with [^35^S]methionine (Figure 4B), respectively. The ratio of both accumulated and nascent GRP78 to actin did not change during rECP treatment. As for positive control, when the cells were treated with 1 μM TG, an ER toxin, a 2- to 4-fold increase in accumulated GRP78 after 4 to 24 h treatment was observed; moreover, newly synthesized GRP78 revealed a 4- to 6-fold increase after 4 to 24 h under the same condition. Taken together, these results implied that rECP-induced apoptosis was ER-independent, in consistence with the results of caspase-12 inhibitor treatment (Figure 3C).

### rECP-induced apoptosis is not mitochondria-dependent

**Figure 5:**
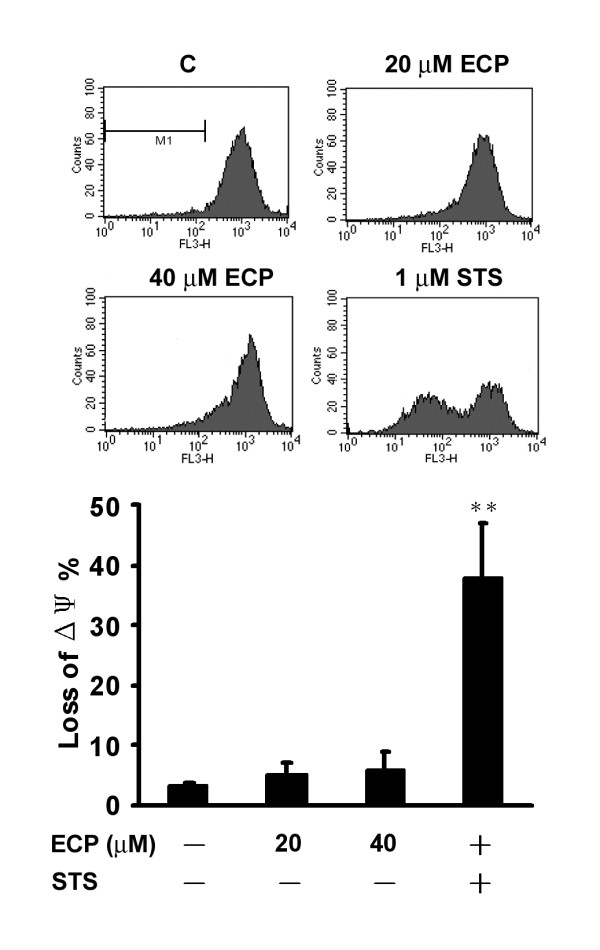
**Effect of rECP-induced apoptosis on MMP**. The MMP of BEAS-2B cells, stained with MitoTracker (Red CMXRos), represents the percentage of the damage in mitochondria. BEAS-2B cells were incubated in the absence or presence of 20 μM rECP, 40 μM rECP or 1 μM staurosporine (STS) for 24 h. After treatment, the MMP of cells was determined by FACS. We gated M1 for calculation of intensity shift. All data represent the arithmetic mean ± SEM. **P < 0.01.

It has been reported that loss of MMP is involved in apoptosis [51]. To investigate whether mitochondrial events were involved in rECP-induced apoptosis, MMP was measured by staining with MitoTracker (Red CMXRos) and analyzed by FACS. BEAS-2B cells treated with 1 μM STS, as a positive control, resulted in 37.9 ± 9% MMP, indicating that approximately 40% of mitochondria were damaged. However, cells treated with 20 and 40 μM rECP revealed MMP values of 5.1 ± 1.8% and 6.1 ± 2.7%, respectively, which did not substantially differ from the untreated control cells (3.3 ± 0.4%) (Figure 5). These results suggested that the caspase-9-dependent mitochondrial apoptotic pathway was not involved in rECP-induced apoptosis, in agreement with the results of caspase-9 inhibitor treatment (Figure 3B). Moreover, cells treated with 10 and 20 μM rECP did not alter cytochrome *c *release, strongly suggesting the notion that rECP did not cause damage in mitochondria (Additional file 2).

### Caspase-8 is involved in rECP-induced apoptosis

**Figure 6:**
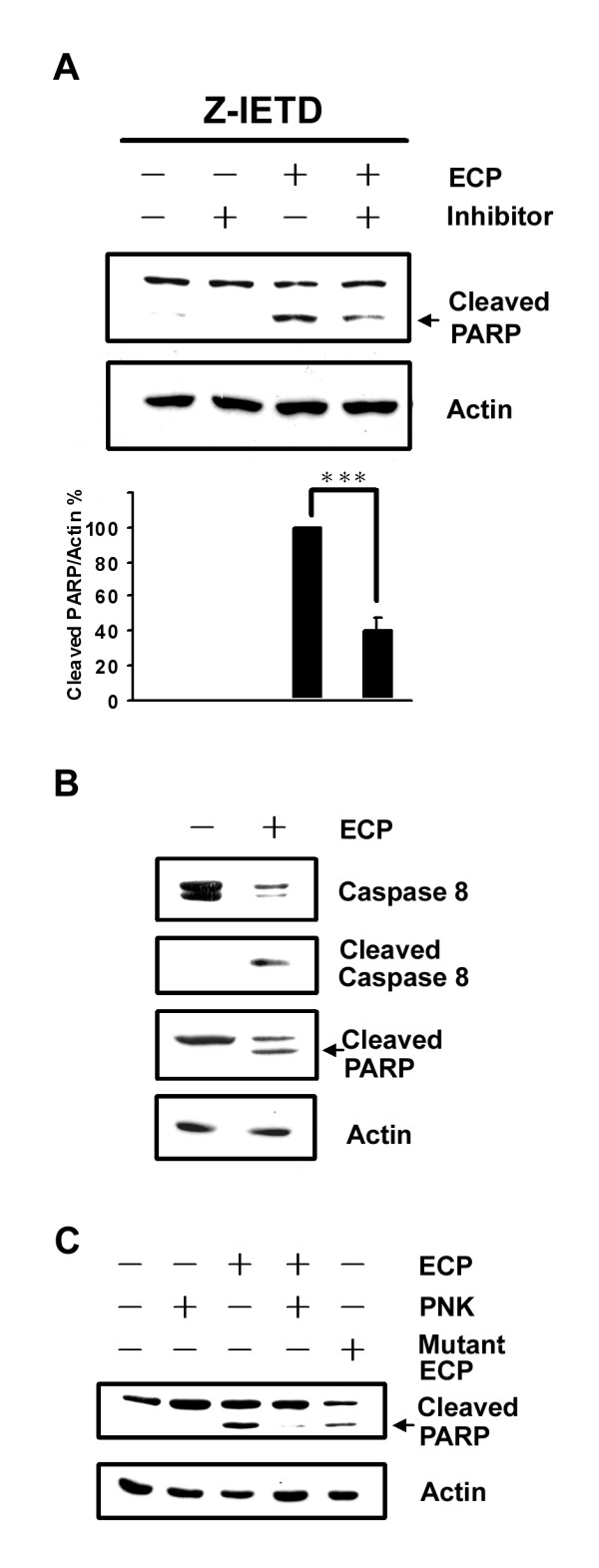
**Effect of caspase-8 on rECP-induced apoptosis**. (**A**) Cells were treated with 20 μM caspase-8 inhibitor (Z-IETD) for 30 min before rECP treatment. After a 48-h incubation in the presence or absence of 20 μM rECP, total cell lysates were resolved by 10% SDS-PAGE. Relative cleaved PARP was assessed by western blotting using anti-PARP and anti-actin followed by quantitative analysis. (**B**) BEAS-2B cells were cultured for 48 h in the presence or absence of 20 μM rECP. Total cell lysates were resolved by 15% SDS-PAGE. Caspase-8 and PARP were analyzed by western blotting using anti-caspase-8 and anti-PARP. (**C**) rECP was incubated with proteinase K (PNK) (250 μg/ml) for 12 h followed by heating in boiling water for 10 min. Cells were treated in the presence or absence of rECP (20 μM), heated PNK (25 μg/ml), heated mixtures (20 μM rECP and 25 μg/ml PNK), and rECP H15A/K38I/H128A mutant (20 μM) for 48 h. All data represent the arithmetic mean ± SEM. ***P < 0.001.

Caspase-8 is a downstream target of the death receptor-initiated pathway [52]. To identify possible involvement of caspases-8 in ECP-induced apoptosis, BEAS-2B cells were treated with rECP in the presence or absence of caspase-8 inhibitor Z-IETD-FMK. The levels of cleaved PARP decreased 40% upon pre-treatment with Z-IETD-FMK (Figure 6A), suggesting that ECP-induced apoptosis proceeded possibly *via *the caspase-8-specific pathway. To examine caspase-8 and PARP activation during rECP-induced apoptosis, BEAS-2B cells were treated with 20 μM rECP for 48 h. The presence of specific cleavage products of caspase-8 and PARP were activated (Figure 6B), suggesting that these precursors were activated and rECP-induced apoptosis was indeed mediated by caspase-8 pathway in BEAS-2B cells.

To confirm that induction of caspase-dependent apoptosis was not derived from lipopolysaccharide, a contaminant often observed in recombinant proteins produced in *E. coli*, rECP was treated with proteinase K (PNK) prior to addition to BEAS-2B cells. It was clear that no PARP cleavage was generated in the presence of either heated rECP or the PNK-treated rECP mixture (Figure 6C), strongly suggesting that apoptosis was indeed induced by rECP itself but not by endotoxins or contaminants in the sample. Moreover, mutant rECP H15A/K38I/H128A (mECP), devoid of the RNase activity, also induced apoptosis, in consistent with the hypothesis that the RNase activity was not essential for cytotoxicity of ECP [13].

### rECP-induced apoptosis is involved in TNF-α response

**Figure 7:**
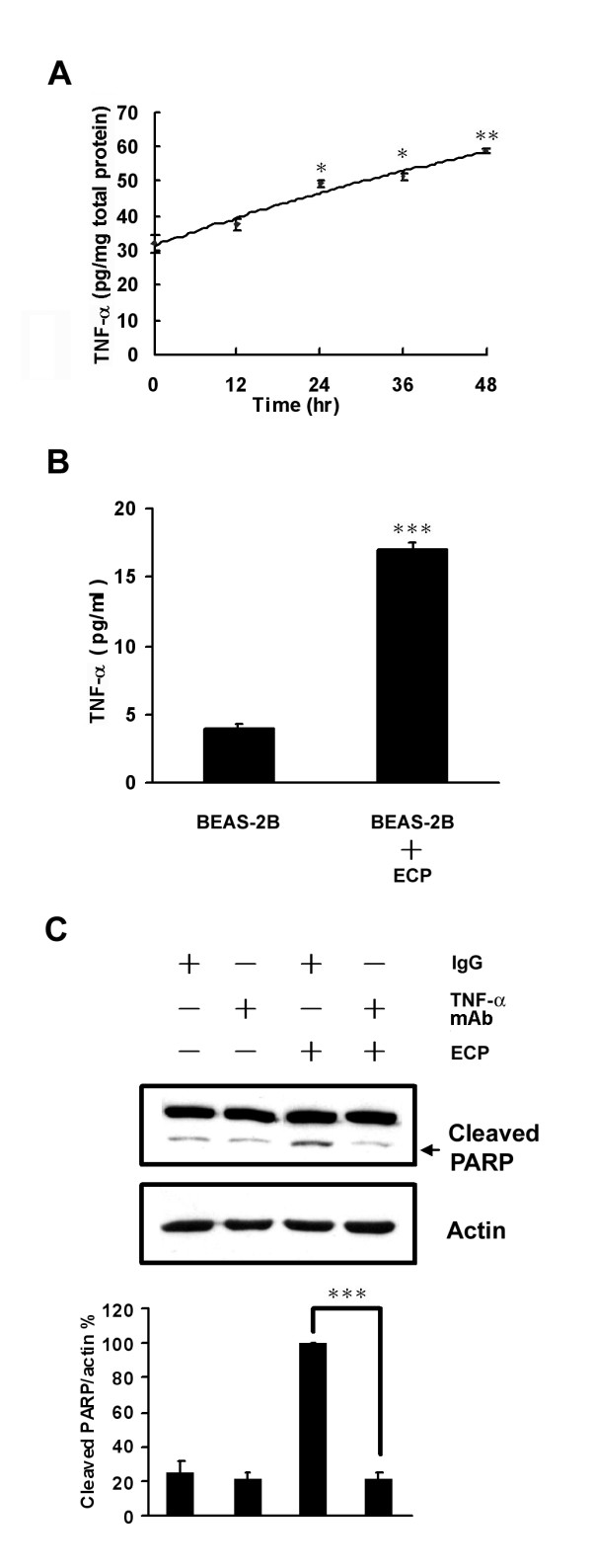
**Effects of TNF-α on rECP-induced apoptosis**. BEAS-2B cells were treated with 20 μM rECP. (**A**) TNF-α was measured in cell lysates by rECP treatment for ranging from 0 to 48 h. (**B**) TNF-α was measured in cultured supernatant medium by rECP treatment for 48 h. All the TNF-α measurements were determined by ELISA assay. (**C**) To investigate the role of TNF-α in rECP-induced apoptosis, BEAS-2B cells were pretreated with 5 μM anti-TNF-α Ab for 4 h before rECP treatment. The addition of IgG Ab to the medium of cells was used as controls in neutralization studies. All data represent the arithmetic mean ± SEM. *P < 0.05, **P < 0.01, ***P < 0.001.

BEAS-2B cells treated with rECP induced TNF-α production and release (Figures 7). Secretion of TNF-α in the culture medium was monitored in BEAS-2B cells treated with rECP for periods from 0 to 48 h (Figure 7A), suggesting that TNF-α production in rECP-treated cells was time-dependent. An ELISA analysis showed that TNF-α accumulation in cell lysate of BEAS-2B cells significantly increased in those treated with rECP after 24 h (49.9 ± 0.5 pg/mg total proteins). The maximum of TNF-α production in the cells reached at 48 h (58.8 ± 0.6 pg/mg total protein). In addition, higher TNF-α level was detected in the supernatant of BEAS-2B cells treated with rECP for 48 h than control cells (16.9 ± 0.1 and 4.0 ± 0.6 pg/ml, respectively) (Figure 7B). In this study, we have found that mutant ECP lacking of RNase activity (mECP) can also induce TNF-α liberation; however, there is no significant increase of TNF-α liberation upon treating with RNase A (Additional file 3).

Previous results showed that eosinophils induced cells to undergo apoptosis accompanying with increasing TNF-α production [26]. To exclude the effect of TNF-α in rECP-induced apoptosis in BEAS-2B cells, an anti-TNF-α antibody (Ab) was used to deplete TNF-α in the culture medium. When BEAS-2B cells were pre-treated with anti-TNF-α Ab, the levels of cleaved PARP significantly decreased to 22% (Figure 7C). Taken together, we have provided the first direct evidence that rECP induced BEAS-2B cells to produce TNF-α, which in turn leads to apoptosis *via *caspase-8 dependent pathway.

## Discussion

AECs play an important role in protecting themselves from external invasion by forming a physical barrier. It has been reported that concentrations of ECP of the sputum is positively correlated with airway inflammation and asthma severity [46], hence higher sputum ECP concentration up to μM level was detected in asthmatic patients [7]. The patches of denuded epithelium were observed in airway biopsies of asthmatic patients [49]. ECP and EDN, having high sequence and structural similarity, are released from activated eosinophils. They inhibit the growth of HL-60 cells (human promyelocytic leukemia cells) [10] and Kaposi's sarcomas cells [53]. Although both ECP and EDN induce apoptosis in cells, the mechanism has not been fully elucidated [21,22]. Recently, ECP was shown to inhibit the viability of BEAS-2B cells as analyzed by MTT assay, but it has never been reported that ECP could cause apoptosis in BEAS-2B cells. Our results of increase in chromatin condensation, sub-G1 population, PARP cleavage, and DNA fragmentation strongly indicate that ECP induces apoptosis in BEAS-2B cells. The study by Trautmann et al. showed that rECP-induced cell death *via *necrosis in bronchial epithelial cells (ECs), which might be attributed to high sensitivity to rECP, and all rECP-induced apoptotic cells activated signals to necrosis in ECs [24]. In addition, the study by Nicotera et al. showed that apoptosis and necrosis death in cell were often intertwines; through apoptosis pathway, caspase activation could cause necrosis by promoting ion overload [54]. Cell type specific response may account for different sensitivity to ECP and different stage of cells in our case. Pre-treatment with general caspase inhibitor impedes ECP cytotoxicity, suggesting that ECP-induced apoptosis is caspase-dependent. It has been known that mitochondrial damage, ER response, and death receptor activation would trigger caspase-dependent apoptosis. Hence three specific caspase (caspase-8, -9 and -12) inhibitors were used to investigate the possible pathways during such caspase-dependent apoptosis. Most apoptosis is linked to mitochondria-related damage, but pre-treatment with a caspase-9 inhibitor did not show any effect in our case. MMP and cytochrome *c *release experiments also confirmed this point. In addition, procaspase-12 cleavage to form active caspase-12 may take place if the ER response has been activated [55]. Although the study shows that human caspase-12 is regarded as a pseudogene because of losing function with several mutations [56], Saleh et al. have reported that caspase-12 shows natural polymorphism in ethnic groups of African descent [57]. In this study, pre-treatment with a caspase-12 inhibitor, metabolite labeling and Western blotting for GRP78 indicated that rECP did not affect the ER response. Apparently rECP-induced apoptosis was not involved in ER response for the protein level of GRP78 was not altered with or without ECP treatment. Therefore, ECP-induced apoptosis was neither caspase-9 nor caspase-12 dependent. Alternatively, the death receptor pathway which undergoes caspase-8 signal transduction, might be involved in ECP-induced apoptosis. Caspase-8-dependent apoptosis may be triggered by cell surface death receptors such as TNFR and Fas...etc. [32]. Till now activation of the caspase-8 pathway in cells treated by eosinophils has never been reported. Recently, ECP was proved to induce apoptosis undergoing caspase-3 like pathway [21]. However, no correlation with caspase-8 has been mentioned. In this study pre-treatment with caspase-8 inhibitor clearly demonstrated that apoptosis was mediated through caspase-8 activation, and cleavage of caspase-8 offered strong evidence to support this notion. This is the first study showing direct correlation between rECP and caspase-8 activation in bronchial epithelial cells, which in turn results in cell apoptosis.

TNF-α or FasL may serve as the death ligands to AECs during caspase-8-dependent apoptosis and TNF-α has been reported to induce apoptosis in AECs [26]. It has also been known that both ECP and TNF-α are released from activated eosinophils [26] Epithelial cells release cytokines and growth factors such as IL-6, TNF-α and TGF-β under environmental stress to remove injured cells and recruit healthy cells [43]. However, there is no report indicating the correlation between rECP and TNF-α liberation. Trautmann et al. found that IFN-γ stimulated eosinophil lysate induced bronchial epithelial cells to undergo apoptosis [24] TNF-α played an important role in IFN-γ stimulated eosinophil-induced apoptosis in bronchial epithelial cells, as evidenced by TNF-α antibody blocking experiment. Besides, previous study showed that co-culture with house dust mite-activated eosinophils and airway bronchial epithelial cells induced TNF-α release; the inhibition experiment further indicated that p38 MAPK and NF-κB were involved in TNF-α release in eosinophil-AECs system [58]. Since ECP is the major component in eosinophils, it is possible that rECP induced TNF-α production may also involve NF-κB and MAPK pathways. Here we hypothesized that up-regulated TNF-α, triggered by rECP treatment, was released to external environment, where it killed cells *via *a feedback mechanism. In this way, the death receptor-triggered pathway would be stimulated to promote apoptosis. As a result, ECP might be recognized by cells as portending pathogen invasion, thereby inducing certain immune responses such as cytokine production and apoptosis. In this study, it found that the inactive RNase, mECP, could still induce TNF-α production, but highly active RNase A showed no significant TNF-α production, strongly suggesting that RNase activity did not correlated with TNF-α production (Additional file 3).

**Figure 8:**
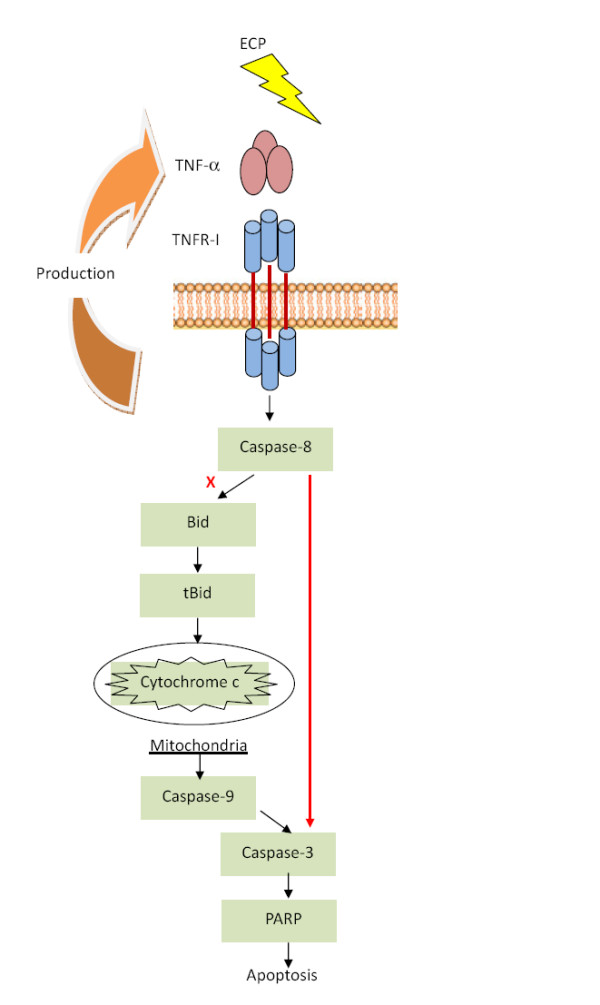
**Role of rECP in TNF-α apoptosis signaling**. rECP increases BEAS-2B cells TNF-α production and release. The release of TNF-α binding to TNF receptor results in receptor internalization and activates caspase-8. Caspase-8-induced apoptosis can either trigger mitochondrial response or directly cause PARP activation by caspase-3. However, rECP-induced apoptosis shows no effects on mitochondrial responses. Accordingly, we suggest that rECP induces mitochondria-independent apoptosis.

TNF-α receptor activation triggered apoptosis can undergo either mitochondria-dependent pathway which is involved in tBid activation and triggers caspase-9 activation by releasing cytochrome *c*, or mitochondria-independent pathway [59]. In our study, caspase-9 inhibitor, MMP assays and cytochrome *c *release experiments all indicated that rECP did not induce mitochondrial response, hence the apoptosis underwent mitochondria-independent pathway. Previous study has reported that caspase-6 is able to activate caspase-8 and involved in mitochondrial response [60]. However, it was proved that ECP-induced apoptosis did not require mitochondrial response; hence we speculated that caspase-8 was activated by TNFR pathway instead of caspase-6. Taken together, Figure 8 presents that ECP induces apoptosis involved in TNF-α-related caspase-8 activation through mitochondria-independent pathway.

Although ECP belongs to the pancreatic-type RNase family [6], its RNase activity is relatively weak. Moreover, the RNase activity of ECP is not essential for its cytotoxicity [13]. ECP, EDN, and RNase A (RNase 1) all belong to the pancreatic RNase family, and their RNase activities can be detected (Additional file 4). As illustrated in our additional file 5, ECP and mutant rECP H15A/K38I/H128A with low or no RNase activity have higher toxicity toward BEAS-2B cells, whereas EDN and RNase A with high RNase activity show no toxicity toward BEAS-2B cells. Accordingly, our study also confirms that rECP lacking of RNase activity retains cytotoxicity. On the contrary, human RNase A is highly enzymatically active in RNA degradation but has no cytotoxicity. Therefore, we suggest that the cytotoxicity of ECP is not correlated to the RNase activity.

Onconase (ONC), one member of bullfrog RNase A superfamily, displays apoptosis to tumor cells *via *caspase-9 dependent but caspase-8 independent pathway [23]. Different from ONC, in this study, ECP triggers apoptosis *via *caspase-8 dependent but caspase-9 independent pathway. Recently, ONC was found to enter cells by clathrin-dependent endocytosis [61]. However, ECP endocytosed into BEAS-2B cells by non-clathrin but lipid raft-dependent macropinocytosis [19]. Accordingly, we speculate that the mechanisms of toxic RNase endocytosis may activate different caspase pathways in target cells.

ECP endocytosis into BEAS-2B cells are facilitated by HSPGs [19]. Interestingly, HS was also detected on the surface of A549 cells (lung carcinoma cells) [62]. Consequently, we found that rECP could induce apoptosis in A549 cells too (data not shown). Taken together, HS plays an important role in toxin endocytosis and triggering apoptosis in lung epithelial cells. Through specific interaction between ECP and HS, ECP can target cancer cells that are rich in HS [63]. Our results suggest that ECP-induced apoptosis might provide novel therapies for specific cancer cells.

## Conclusions

In summary, we found that rECP could inhibit BEAS-2B cell viability and induce apoptosis. Increase of TNF-α in cells and medium, as well as cleavage of caspase-8 in BEAS-2B cells were detected after rECP treatment. However, neither MMP nor ER response was observed in the rECP-induced apoptotic cells. In addition, caspase-9 and -12 inhibitor assays confirmed such speculation. Thus, we clearly demonstrate that rECP causes BEAS-2B cell apoptosis mainly through TNF-α-mediated caspase-8 specific pathway in a mitochondria-independent manner. The knowledge of this molecular basis is pivotal in understanding the development of pathogenesis in asthma and shed a light on potential therapeutic applications.

## Methods

### Cell and cell culture

BEAS-2B cells purchased from American Type Culture Collection (ATCC) were maintained in RPMI-1640 medium (Sigma-Aldrich) supplemented with heat-inactivated 10% fetal calf serum (Gibco/Invitrogen), 100 U/ml penicillin, and 100 μg/ml streptomycin sulfate. Cells were maintained in 9-cm culture dishes with 5% CO_2 _at 37°C. Cells were sub-seeded in appropriate culture vessels (6- or 12-well plates) and incubated at 37°C for 24 h prior to all treatments.

### Expression and purification of rECP and mutant ECP

Both recombinant ECP (rECP) and H15A/K38I/H128A mutant rECP (mECP) were expressed in *Escherichia coli *and purified as described [64] with minor modification. rECP and mECP containing a *C*-terminal His_6 _tag were expressed in *E. coli *BL21(DE3) (Novagen) and purified by affinity column chromatography (BD Biosciences). For each preparation, 10 ml of overnight culture was inoculated into 1 L LB containing 100 μg/ml ampicillin, and grown at 37°C for 6 h. Isopropyl-β-D-thiogalactopyranoside (IPTG, Sigma-Aldrich) was added to a final concentration of 0.5 mM, and after induction at 37°C for 6 h, the *E. coli *was harvested by centrifugation at 3000 rpm. rECP and mECP were collected from inclusion bodies that were refolded by dialysis in refolding buffer (20 mM Tris, 0.5 M arginine, 0.2 mM GSSG, 2 mM EDTA, 10% glycerol, pH 8.5). The purified rECP and mECP were concentrated by Amicon Ultra-15 (Millipore) and stored in phosphate-buffered saline (PBS) at -80°C until use. After purification, we used Endotoxin Removing Gel (Pierce) to remove LPS before rECP storage and also checked LPS residual level by HEK-Blue™ LPS Detection Kit (InvivoGen) before each treatment.

### MTT cell viability assay

The toxicity effect of rECP on cell viability was determined by a colorimetric assay using 3-(4,5-dimethylthiazol-2-yl)-2,5-diphenyltertrazolium bromide (MTT) (Sigma-Aldrich) as described [65]. Briefly, cells (5,000~10,000) were seeded in 96-well plate and incubated overnight at 37°C, 5% CO_2_. The cells were treated with the indicated concentration (up to 40 μM) of rECP. After treatment with rECP for 48 h, 10 μl MTT (5 mg/ml in PBS) was added to 90 μl of culture medium/well for 4 h. Levels of MTT were determined by measuring the absorbance at 570 nm.

### Detection of chromatin condensation

Hoechst 33342 (1 μg/ml) (Sigma-Aldrich) was added 20 min prior to the end of the incubation period in the dark. The cells were washed with cold PBS twice and fixed with 3.7% formaldehyde for 15 min. The nuclei of apoptotic cells were observed by fluorescence microscopy.

### Detection of apoptosis by sub-G1 fractions and Annexin-V-FITC

To determine the sub-G1 fractions, detached BEAS-2B cells were fixed in 75% ethanol at -20°C overnight and centrifuged at 1000 × *g *for 5 min to remove ethanol, followed by treatment with 50 μg/ml RNase A and staining with 50 μg/ml propidium iodide (PI) (Sigma-Aldrich) on ice for 30 min in the dark [66]. The stained cells were analyzed by fluorescence-activated cell sorting (FACS) (Becton Dickinson) according to the manufacturer's instructions. The levels of early apoptosis were determined using the Annexin-V-FITC Apoptosis Detection kit (BD Biosciences). The method was performed as described [67] with minor modification. After trypsinization, BEAS-2B cells were washed twice with cold PBS and resuspended in 1× binding buffer. The cell suspension (200 μl) was transferred to 5-ml tubes, and 5 μl annexin V was added. After incubation with annexin V for 5 min at 4°C, 5 μl PI was added. The cells were incubated at 4°C in the dark for 15 min, and 800 μl of binding buffer (10 mM HEPES, pH 7.4, 140 mM NaCl, 2.5 mM CaCl_2_) was added before FACS analysis.

### Detection of mitochondrial membrane potential (MMP)

To determine MMP, the detached BEAS-2B cells were stained with 100 nM MitoTracker Red CMXRos (Molecular Probes) in RPMI medium for 20 min at 37°C in the dark. After typsinization, the stained cells were analyzed by FACS. Fluorescence of PI was collected in the FL2 or FL3 detector, and fluorescence of MitoTracker was collected in the FL3 detector. All data were evaluated using Cell Quest software (Becton Dickinson).

### Caspase and TNF-α inhibitors treatment

Benzyloxycarbonyl-Val-Ala-Asp-fluoromethylketone (Z-VAD-FMK) (Calbiochem), benzyloxycarbonyl-Ile-Glu-Thr-Asp-fluoromethylketone (Z-IETD-FMK) (Calbio-chem), benzyloxycarbonyl-Leu-Glu(OMe)-His-Asp(OMe)-fluoromethylketone (Z-LE(OMe)HD(OMe)-FMK) (Calbiochem), and benzyloxycarbonyl-Ala-Thr-Ala-Asp-fluoromethylketone (Z-ATAD-FMK) (BioVision) are irreversible cell-permeable inhibitors of the general caspase pathway, caspase-8-, caspase-9- and caspase-12-specific pathways, respectively [68]. For studies concerning the effect of inhibitors, each inhibitor (stored in 10 mM DMSO) was added to BEAS-2B cells at 20 μM for 30 min prior to the addition of rECP.

For the TNF-α inhibitor studies, BEAS-2B cells were treated with rECP neutralized with/without anti-TNF-α antibody (Ab) (5 μg/ml; Abcam). The addition of polyclonal rabbit IgG Ab (5 μg/ml; Sigma-Aldrich) to the medium of cells was used as controls in inhibitor studies. The dose of the inhibitors was used in the information based on the efficacy in the inhibition of the activity of the cytokines but not cause cytotoxicity (trypan blue dye exclusion).

### Western blotting

BEAS-2B cells treated with rECP neutralized with/without the inhibitors. Cell lysates were homogenized by sample buffer (100 mM Tris-HCl (pH 6.8), 2% Sodium Dodecyl Sulfate (SDS), 0.002% bromophenol blue, 20% glycerol, 10% β-mercaptoethanol (all from Sigma-Aldrich). Those were subjected to SDS-PAGE and transferred onto nitrocellulose membranes (Amersham Biosciences). The following primary antibodies were used for immunodetection: rabbit anti-human poly(ADP-ribose) polymerase (PARP) (Cell Signaling), goat anti-human actin (Santa Cruz Biotechnology), mouse anti-human caspase-8 (Calbiochem), and rat anti-human GRP78 (Santa Cruz Biotechnology). Secondary antibodies conjugated to horseradish peroxidase (Santa Cruz Biotechnology) and the Western Blot Substrate kit (Pierce) were used to detect chemiluminescence.

### De novo protein synthesis

Metabolic labeling of nascent proteins was conducted as described [69]. At the end of various treatment periods, the cells were washed with PBS twice, and replaced with RPMI medium containing [^35^S]methionine (20 μCi/ml) for 2 h. After removal of the medium, the cells were washed with PBS twice and lysed with 2× sample buffer. Equal amounts of cells were heated at 100°C in sample buffer for 10 min and resolved by SDS-PAGE. The gel was dried for 2 h and exposed to X-ray film for 4 days before development.

### Quantitative measurement of TNF-α

For determination of cell-associated cytokine concentrations, cell lysate was prepared using protein extract buffer containing 0.6 M KCl, 1% Triton X-100, 0.02 M Tris-HCl (pH7.0), 1.0 mM phenylmethylsufonyl fluoride, and 50 μg/ml aprotinin (all from Sigma-Aldrich). After centrifugation at 9,500 *g *for 3 min at 4°C, protein samples in the supernatant were immediately transferred to a clean tube, and the concentration assessed using DC protein assay kit (Bio-Rad). Supernatant and lysate TNF-α concentrations were determined using corresponding ELISA Ready-SET-Go kits (eBioscience) and expressed in pictograms of TNF-α per milligram of cellular protein. The optical density was detected using a VERSAmax microplate reader (Molecular Devices) and the levels of each cytokine were deduced from the absorbance value by extrapolation from a standard curve generated in parallel.

### DNA fragmentation

DNA fragmentation assay was conducted as described [70] with minor modification. Cells were washed twice in cold PBS and resuspended in 100 ml of lysis buffer (10 mM Tris-HCl, pH8, 1 mM EDTA, pH 8.0, 0.5%N-lauroyl sarcosine, 0.02 mg/ml RNAse A, and 0.25 mg/ml proteinase K). After incubation for 10 min at 55°C, the sample was loaded into the 2% agarose gel. Electrophoresis was then performed in TBE buffer (89 mM Tris-base, 89 mM boric acid, 2 mM EDTA pH 8.0).

### Cytochrome C release detection

Cytochrome C release detection assay was followed as described [70] with minor modification. Cells were put on ice for 10 min and washed twice in cold PBS. Cell pellets were then resuspended in 250 ml of buffer A (75 mM KCl, 1 mM Na_2_HPO_4_, 8 mM Na_2_HPO_4_, 250 mM sucrose, 230 mg/ml digitonin) and incubated on ice for 10 min. After centrifugation at 15,000 g for 10 min at 4°C, supernatant were kept to obtain the cytosolic fractions. The cytosolic fraction was mixed with an equal volume of 2× RIPA buffer (1× RIPA is 1% IGEPAL, 0.5% sodium deoxycholate, 0.1% SDS, 0.2 mM sodium orthovanadate, 50 mM sodium fluoride, 0.1 mg/ml phenyl methyl sulfonyl fluoride).

### Ribonuclease assay

The RNase activity of the recombinant proteins against a yeast tRNA (Sigma-Aldrich) substrate was measured in 20 mM Tris-HCl buffer (pH 8.0) at 37°C. Purified RNase (30 pmol) was added into 50 μl of the Tris buffer with 120 μg of tRNA. The reaction was stopped by addition of 200 μl 0.7% perchloric acid with 0.1% uranyl acetate and incubated on ice for 30 min. The insoluble tRNA was removed by centrifugation at 14,000 *g *for 15 min at 4°C. The amount of solubilized tRNA was determined by UV absorbance at 260 nm. The catalytic activity of the RNase was determined as the nanogram of RNA digested per second per nanomol of RNase used.

### Statistical analysis

Results were described as mean ± standard deviation. All statistical analysis was conducted by the statistical package SPSS13.0. The differences were investigated using Student's *t*-test and one-way analysis of variance (ANOVA). Values of *P *are considered to be statistically significant: *P < 0.05, **P < 0.01; ***P < 0.001.

## List of abbreviations

Ad12SV40: adenovirus 12-SV40 virus hybrid; AECs: airway epithelium cells; ECP: eosinophil cationic protein; rECP: recombinant ECP; mECP: H15A/K38I/H128A mutant rECP; EDN: eosinophil derived neurotoxin; ER: endoplasmic reticulum; FACS: fluorescent-activated cell sorting; GRP78: 78-kDa glucose regulated protein; HS: heparan sulfate; HSPG: heparan sulfate proteoglycan; MMP: mitochondrial membrane potential; MTT: 3-(4,5-dimethylthiazol-2-yl)-2,5-diphenyltertrazolium bromide; ONC: onconase; PARP: poly (ADP-ribose) polymerase; pI: isoelectric point; PI: propidium iodide; PNK: Proteinase K; RNase: ribonuclease; STS: staurosporin; TG: thapsigargin; TNF-α: tumor necrosis factor-alpha; TNFR1: TNF receptor-1; TRADD: TNFR-associated death domain; TRAIL: TNF-related apoptosis inducing ligand; Z-LE(OMe)HD(OMe)-FMK: benzyloxycarbonyl-Leu-Glu-(OMe)-His-Asp(OMe)-fluoromethylketone Z-IETD-FMK: Benzyloxycarbonyl-Ile-Glu-Thr-Asp-fluoromethylketone; Z-VAD-FMK: Benzyloxycarbonyl-Val-Ala-Asp-fluoromethyl-ketone; Z-ATAD-FMK: benzyloxycarbonyl-Ala-Thr-Ala-Asp-fluoro-methylketone.

## Authors' contributions

YKL and MDTC originated the project, supervised the overall conduct of the research. KCC and CWL carried out major experimental work in this study and drafted the manuscript. TCF and CWS performed data analysis. CHC assisted TNF-α detection. CTC helped rECP purification. SLF helped FACS experiment and data analysis. CCC participated in manuscript preparation. JJT adviced manuscript drafting. All authors read and approved the final manuscript.

## Supplementary Material

Additional file 1 **Supplementary Figure 1. rECP induces DNA fragmentation in BEAS-2B cells**. BEAS-2B cells (5 × 10^5^) were incubated in a 10 cm dish in the absence or presence of 20 μM of rECP for 48 h. DNA damage indicating apoptosis was determined by the DNA fragmentation. Click here for file

Additional file 2 **Supplementary Figure 2. Effect of cytochrome c release on rECP treatment**. BEAS-2B cells (5 × 10^4^) were incubated in a 6 well plate in the absence or presence of 10, 20 μM of rECP for 24 h. The cytosolic cytochorme c was detected by western blotting. Click here for file

Additional file 3 **Supplementary Figure 3. Effects of TNF-α liberation on various RNases**. BEAS-2B cells were treated with 20 μM RNase A, rECP and mECP. TNF-α was measured in cell lysates by treatment for 48 h. All the TNF-α measurements were determined by ELISA assay. All data represent the arithmetic mean ± SEM. *P < 0.05 Click here for file

Additional file 4 **Supplementary Figure 4. RNase activities of recombinant eosinophil RNases degrading yeast tRNAs**. The RNase activities of rECP and EDN were measured employing a standard assay with yeast tRNA as the substrate, and RNaseA as a positive control. The values indicate in nmol tRNA digested per pmol enzyme per second. Click here for file

Additional file 5 **Supplementary Figure 5. Effect of different ribonucleases on cytotoxicity of BEAS-2B cells**. Equal mounts of cells were cultured in 12-well plates in the presence of 20 μM of rECP, mutant rECP, rEDN and RNase A for 48 h. The cleavage of PARP was detected by western blotting. *P < 0.05 Click here for file
