# Supplementary figures and images for: TNF-α Mediates Eosinophil Cationic Protein-induced Apoptosis in BEAS-2B Cells
Source: BMC Cell Biol. 2010 Jan 20;11:6. doi: 10.1186/1471-2121-11-6 (PMC2819994; doi:10.1186/1471-2121-11-6)

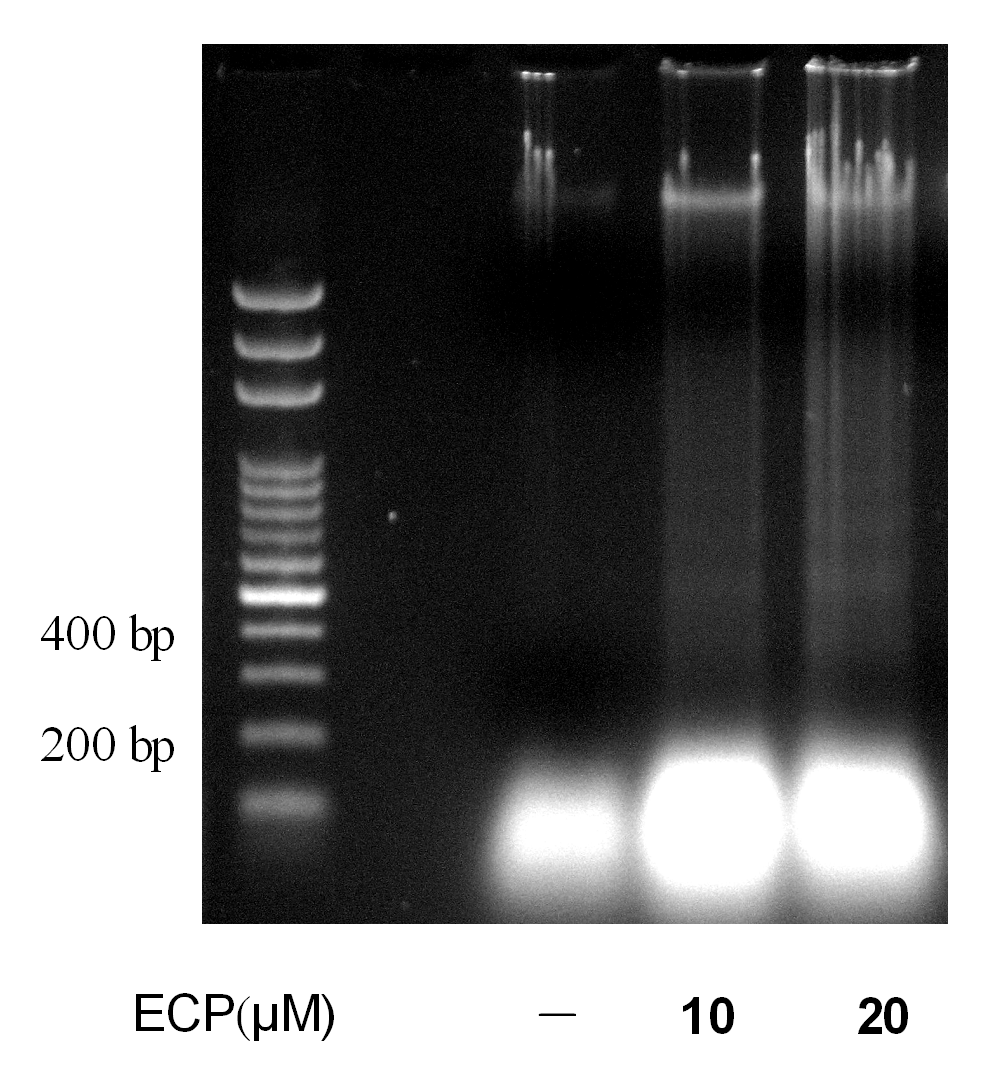

Supplement: Additional file 1 — Supplementary Figure 1. rECP induces DNA fragmentation in BEAS-2B cells. BEAS-2B cells (5 × 10^5^) were incubated in a 10 cm dish in the absence or presence of 20 μM of rECP for 48 h. DNA damage indicating apoptosis was determined by the DNA fragmentation. [file 1471-2121-11-6-S1.TIFF]

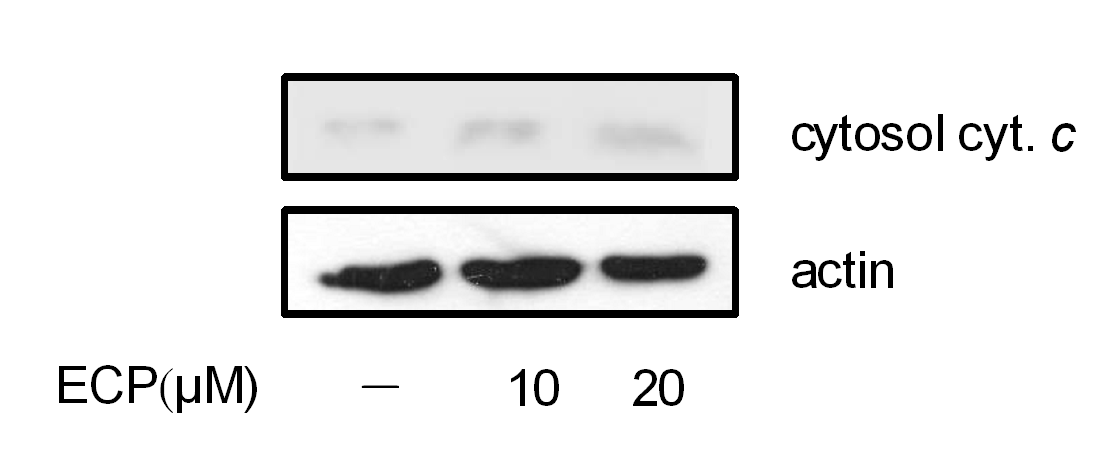

Supplement: Additional file 2 — Supplementary Figure 2. Effect of cytochrome c release on rECP treatment. BEAS-2B cells (5 × 10^4^) were incubated in a 6 well plate in the absence or presence of 10, 20 μM of rECP for 24 h. The cytosolic cytochorme c was detected by western blotting. [file 1471-2121-11-6-S2.TIFF]

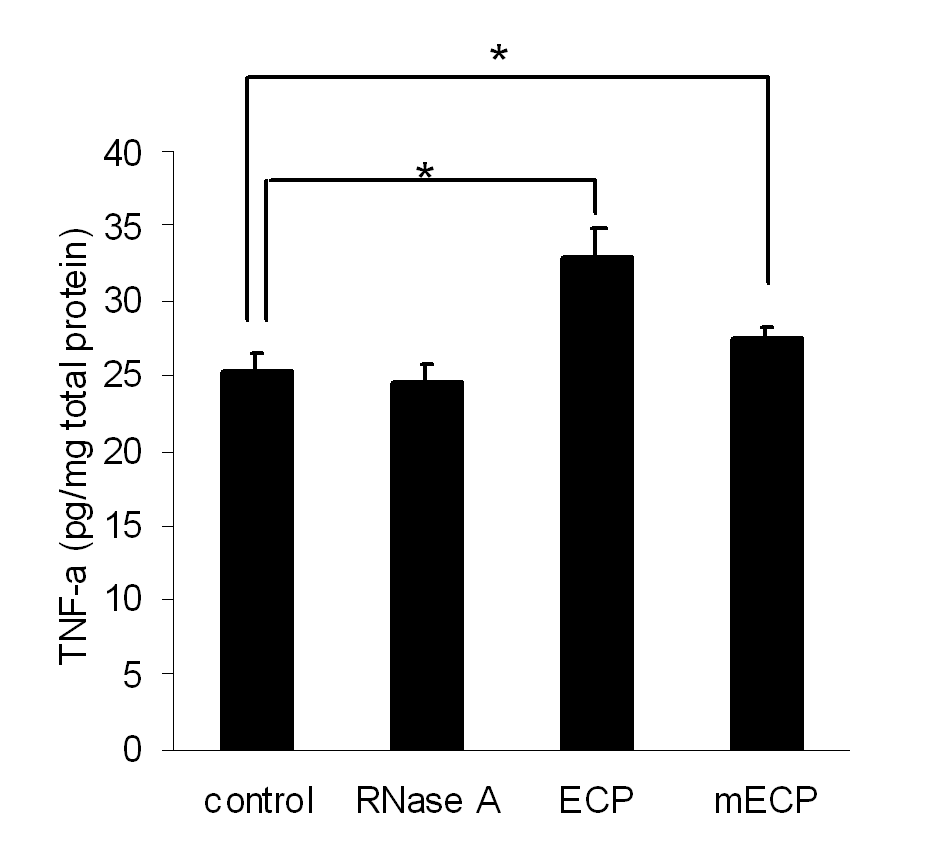

Supplement: Additional file 3 — Supplementary Figure 3. Effects of TNF-α liberation on various RNases. BEAS-2B cells were treated with 20 μM RNase A, rECP and mECP. TNF-α was measured in cell lysates by treatment for 48 h. All the TNF-α measurements were determined by ELISA assay. All data represent the arithmetic mean ± SEM. *P < 0.05 [file 1471-2121-11-6-S3.TIFF]

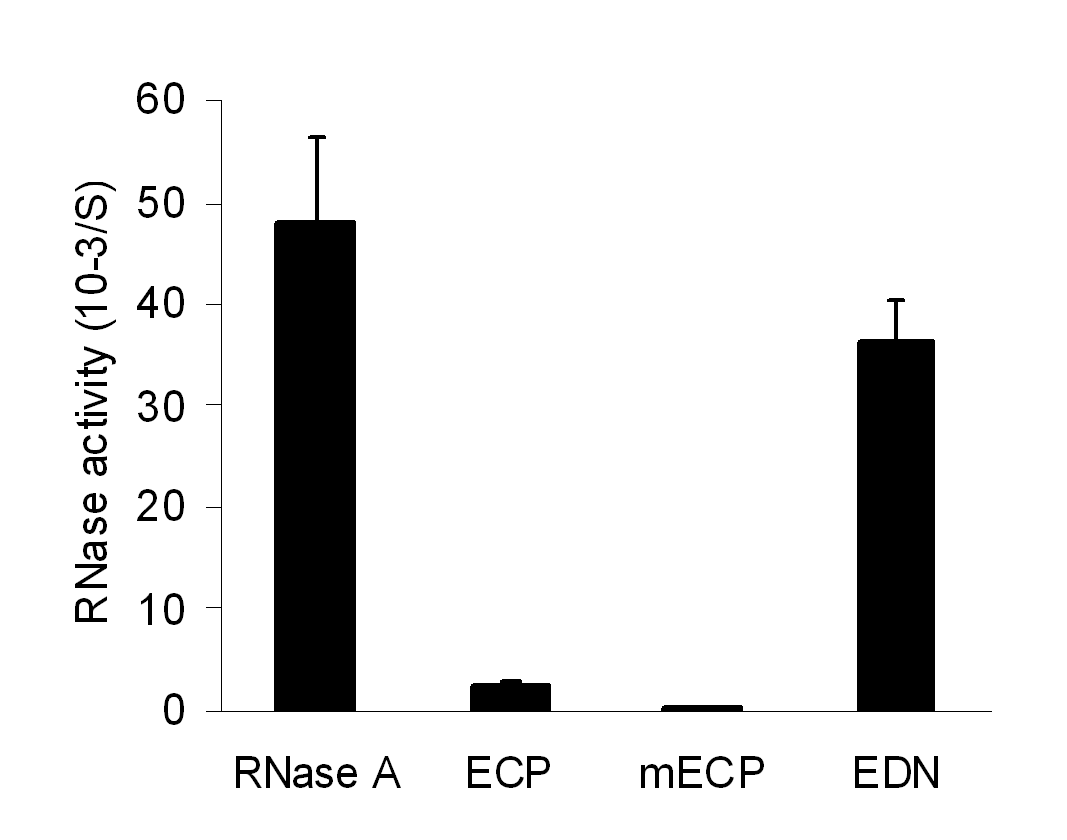

Supplement: Additional file 4 — Supplementary Figure 4. RNase activities of recombinant eosinophil RNases degrading yeast tRNAs. The RNase activities of rECP and EDN were measured employing a standard assay with yeast tRNA as the substrate, and RNaseA as a positive control. The values indicate in nmol tRNA digested per pmol enzyme per second. [file 1471-2121-11-6-S4.TIFF]

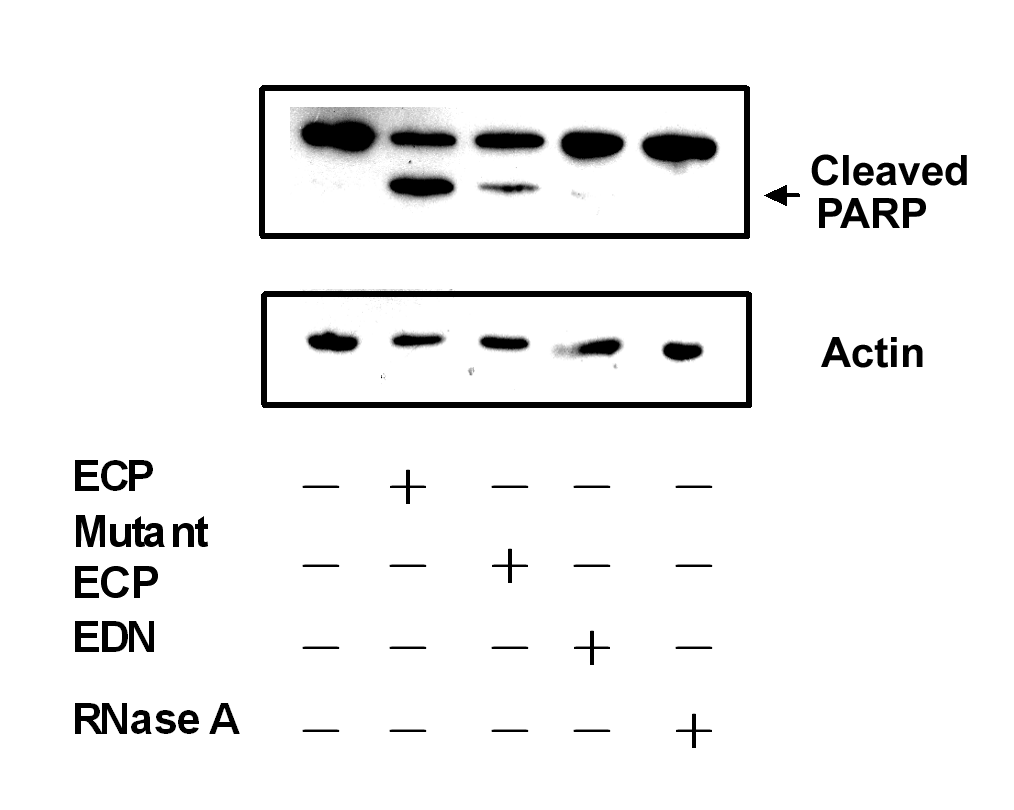

Supplement: Additional file 5 — Supplementary Figure 5. Effect of different ribonucleases on cytotoxicity of BEAS-2B cells. Equal mounts of cells were cultured in 12-well plates in the presence of 20 μM of rECP, mutant rECP, rEDN and RNase A for 48 h. The cleavage of PARP was detected by western blotting. *P < 0.05 [file 1471-2121-11-6-S5.TIFF]
